# Seroprevalence of *Toxoplasma gondii*, Rubella Virus, Cytomegalovirus, and Herpes Simplex Virus Among Children and Women in Bhutan's National Referral Hospital: A Cross‐Sectional Study

**DOI:** 10.1002/puh2.70367

**Published:** 2026-09-19

**Authors:** Karma Norbu, Sonam Wangzin Rabjay, Karma Galey, Thinley Dorji, Ugyen Chophel, Jamyang Wangmo, Binita Rai, Yeshey Dorjey, Tshokey Tshokey

**Affiliations:** ^1^ Department of Health Services Ministry of Health Thimphu Bhutan; ^2^ Department of Emergency Medicine Eastern Regional Referral Hospital Mongar Bhutan; ^3^ Department of Internal Medicine Central Regional Referral Hospital Gelephu Bhutan; ^4^ Faculty of Postgraduate Medicine Khesar Gyalpo University of Medical Sciences of Bhutan Thimphu Bhutan; ^5^ Department of General Practice Wangdue Hospital Wangdue Phodrang Bhutan; ^6^ Department of Pathology and Laboratory Medicine Jigme Dorji Wangchuck National Referral Hospital Thimphu Bhutan; ^7^ Department of Obstetrics and Gynaecology Eastern Regional Referral Hospital Mongar Bhutan

**Keywords:** antibodies, cytomegalovirus, herpes simplex virus, prevalence, rubella, seropositivity, *Toxoplasma gondii*

## Abstract

**Background:**

Congenital infections caused by *Toxoplasma gondii*, rubella virus, cytomegalovirus, and herpes simplex virus (TORCH) infections remain an important but under‐documented contributor to adverse maternal and neonatal outcomes in low‐ and middle‐income countries, including Bhutan.

**Objectives:**

This study aimed to determine the seroprevalence of TORCH infections among children and women in Bhutan's national referral hospital.

**Methods:**

A retrospective cross‐sectional review of TORCH antibody test records was conducted at the Jigme Dorji Wangchuck National Referral Hospital for tests carried out between January 2019 and December 2022. The study included all children <18 years and adult women. Serological testing used rapid immunochromatographic assays (OnSite TORCH Panel) to detect immunoglobulin G (IgG) and immunoglobulin M (IgM) antibodies, and data were analyzed descriptively with subgroup comparisons by age.

**Results:**

A total of 624 participants were included, comprising 359 children (<18 years) and 265 adult females. Among children, 202 (56.9%) were male, and 153 (43.1%) were female. Seropositivity for TORCH infections varied across age groups, with the highest IgG and IgM positivity observed in neonates and infants and a subsequent rise in the reproductive age group. Herpes simplex virus (HSV) showed the highest overall seropositivity (83.8%), followed by cytomegalovirus (CMV) (82.7%), rubella (65.7%), whereas *T. gondii* had the lowest (14.7%). CMV had the highest IgM (4.8%) and combined IgG/IgM positivity (4.3%).

**Conclusions:**

TORCH seropositivity varied across age groups, with HSV and CMV being the most frequently detected infections and IgG antibodies predominating. These findings provide the first baseline TORCH seroprevalence data in Bhutan, which can serve as a reference for future maternal and child health programs.

## Graphical abstract

Congenital infections caused by *Toxoplasma gondii*, rubella virus, cytomegalovirus, and herpes simplex virus were assessed in 624 children and women at JDWNRH (2019–2022). HSV (83.8%) and CMV (82.7%) were most prevalent, whereas *T. gondii* was the lowest (14.7%). Findings provide baseline seroprevalence data for Bhutan and support maternal‐child health surveillance.

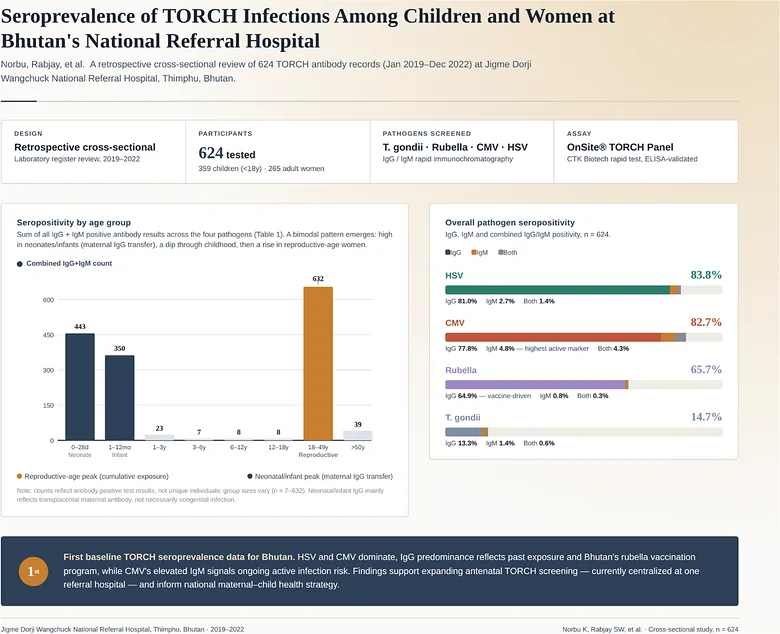

## 1 Introduction

The term TORCH is an acronym for *Toxoplasma gondii* (*T. gondii*), other agents (such as syphilis, human immunodeficiency virus, hepatitis viruses, varicella‐zoster virus [VZV], and parvovirus B19), rubella, cytomegalovirus (CMV), and herpes simplex virus (HSV) [1]. These infections are significant contributors to congenital infections worldwide, especially causing the greatest burden in low‐ and middle‐income countries where access to screening and care are limited [1, 2, 3, 4]. These pathogens can lead to severe adverse outcomes in neonates, including miscarriage, stillbirth, intrauterine growth restriction, preterm birth, congenital anomalies, and long‐term developmental impairments. The risk of congenital infection is strongly influenced by the timing of maternal infection, with infections during the first trimester posing the highest risk for fetal complications [5, 6, 7].

Globally, congenital TORCH infections contribute substantially to neonatal morbidity and mortality. An estimated 2.3 million newborns died within the first 28 days of life in 2022, nearly half due to infectious causes, with TORCH pathogens playing a key role [2, 3, 8]. Because maternal infections are often asymptomatic and clinical presentations overlap, serological testing for immunoglobulin G (IgG) and immunoglobulin M (IgM) antibodies remains the mainstay for diagnosis [4].

*T. gondii* can cause miscarriage, neurological defects, and developmental delays, with marked geographical variation in seroprevalence. A systematic review reported that IgG seroprevalence averages 25.7%, with the highest in Africa (61.4%) and the lowest in Asia (16.4) [9]. Other meta‐analyses have similarly demonstrated high seroprevalence in Africa (51%) and South America (49%) [10], whereas another reported the highest prevalence in Australia (54%), followed by South America (45%) and Africa (42%) [11]. These variations likely reflect differences in climate, dietary practices, sanitation, socioeconomic conditions, and exposure to cats, the definitive host of *T. gondii*.

Rubella virus infection during early pregnancy can cause congenital rubella syndrome (CRS), resulting in heart defects, cataracts, and hearing loss. It is reported that about 9.4% of pregnant women lack rubella antibodies, meaning they are still susceptible to infection [12]. CMV is the leading cause of congenital viral infection worldwide, responsible for sensorineural hearing loss, microcephaly, and developmental delays. Global seroprevalence is estimated at 83% in the general population and 86% among women of childbearing age, with the highest rates in the World Health Organization (WHO) Eastern Mediterranean Region (90%) and the lowest in the WHO European Region (66%) [13]. Congenital HSV infection, though less common, is associated with high morbidity and mortality [14], manifesting as encephalitis, disseminated infection, and neonatal death, with an estimated 11% in people aged 15–49 years [15].

In the South Asia region, a 7‐year hospital‐based survey of 4044 samples in North India reported an overall TORCH seropositivity rate of 33.5%, with the highest burden in women aged 15–25 years (43.1%). Among IgM‐positive cases, HSV accounted for the highest rate (52%), followed by CMV (41%), indicating a significant proportion of active infection [16]. Another hospital‐based study among first‐trimester pregnant women in India found the highest IgM seropositivity for CMV (34.7%) and HSV‐2 (33.5%), followed by rubella (30.4%) and *T. gondii* (19.4%) [17]. Data from Nepal show that HSV‐2 and CMV are the most frequent acute infections [18]. These findings highlight the risk of primary infections during early pregnancy, when the fetus is most vulnerable.

In 2023, Bhutan recorded a neonatal mortality rate of 6.9, an infant mortality rate of 15.2, and an under‐five mortality rate of 19.5 per 1000 live births [19]. Neonatal mortality accounts for over half of under‐five deaths in Bhutan, yet there is limited local data on its underlying causes [20]. The factors driving this high mortality have not yet been adequately explored in Bhutan. Although national data are limited, TORCH infections likely contribute significantly to adverse neonatal outcomes. Despite the availability of free healthcare services, including antenatal care, undiagnosed congenital infections may still play an important role in neonatal morbidity and mortality [21, 22].

This study aimed to generate baseline seroprevalence data on *T. gondii*, rubella, CMV, and HSV among children and adult female patients in Bhutan. The findings will inform maternal‐child health programs, guide future surveillance, and support targeted interventions.

## 2 Methods

### 2.1 Study Design

This was a cross‐sectional study involving a retrospective review of hospital laboratory records from the Microbiology Unit of Jigme Dorji Wangchuck (JDW) National Referral Hospital. Data were collected for all TORCH serological tests conducted between January 01, 2019, and December 31, 2022.

### 2.2 Study Setting

The study was conducted at the JDW National Referral Hospital, Bhutan's apex and national referral hospital, located in Thimphu, the capital city. Thimphu is located in the western part of the country and has the largest population, with 138,736 inhabitants, accounting for 19.1% of Bhutan's total population of 735,553 [23].

The JDW National Referral Hospital not only attends to patients from Thimphu but also from all other parts of the country. On average, the annual outpatient attendance was approximately 568,005, with 17,855 inpatient admissions in 2022 [24]. The hospital also serves as the central hub for specialized diagnostic services, with 993,350 laboratory tests performed annually, which include testing for TORCH. The Microbiology Unit of the JDW National Referral Hospital receives blood samples for TORCH screening across the country [24].

### 2.3 Study Population

The study included all children (<18 years) and adult female patients who underwent TORCH testing at the JDW National Referral Hospital between January 1, 2019, and December 31, 2022. Adult males were excluded, as the focus of the study was maternal and child health and considering that males were not usually tested. Records with missing or incomplete TORCH panel results were also excluded.

### 2.4 Sample Size and Sampling Method

A census of available records was performed, including all eligible patients during the study period. No formal sample size calculation was conducted. This approach ensured complete coverage of available laboratory data and minimized sampling bias.

### 2.5 Laboratory Testing

Sample collection, storage, processing, and testing details were gathered from the Serology Laboratory of the Microbiology Unit where they were performed. Blood samples were collected from both outpatient and inpatient settings and transported to the laboratory under standard biosafety protocols. Serum was separated by centrifugation and stored at 2–8°C until testing. Depending on the workflow, samples were analyzed singly or in batches.

Testing for TORCH infections was performed using rapid immunochromatographic test (ICT) kits called OnSite TORCH Panel Rapid Test (R0253C), CTK Biotech, Inc. China (https://www.ctkbiotech.com), which detects both IgM and IgG antibodies. According to the manufacturer's information, the proportion of results that agree with ELISA reference, the reported accuracy for IgG and IgM detection, was as follows—*T. gondii*: 94.9% for IgG and 98.8% for IgM, Rubella virus: 97.7% for IgG and 96.0% for IgM, CMV: 93.4% for IgG and 93.9% for IgM, HSV‐1: 90.7% for IgG and 85.0% for IgM, and HSV‐2: 95.3% for IgG and 95.2% for IgM.

The test was considered valid only in the presence of a visible control (C) line. The presence of both the C line and M line was interpreted as a positive result for IgM antibodies to the respective pathogen. The presence of both the C line and G line was interpreted as a positive result for IgG antibodies. The presence of C, M, and G lines simultaneously was interpreted as evidence of both IgM and IgG antibodies. The presence of only the C line, with no visible M or G line, was interpreted as a negative result. Equivocal or indeterminate results were repeated using the same serum sample. If results remained inconclusive, a new specimen was collected, and retesting was performed.

### 2.6 Study Variables, Data Sources, and Collection Method

All data were extracted from the Serology Laboratory Register at the Microbiology Unit of the hospital. The variable included age, gender, status of TORCH infections, and seroconversion. Potential duplicate entries (e.g., repeated testing) were identified and excluded on the basis of citizenship Identity number, hospital registration number, and patient names.

### 2.7 Data Analysis and Statistics

Data were entered into Microsoft Excel 2016, and statistical analysis for frequencies, percentages, and subgroup comparisons was conducted using STATA version 18 (StataCorp LP, USA). Descriptive statistics (frequencies, percentages, and medians) were calculated for different variables.

## 3 Results

A total of 675 specimens were received for TORCH testing during the study period. Of these, data from 624 participants were included in the final analysis, whereas 51 were excluded due to incomplete data. Among the 624 participants, 359 (57.5%) were children aged <18 years, and 265 (42.5%) were adult females. Of the 359 children, 202 (56.9%) were male, and 153 (43.1%) were female; sex information was missing for four participants.

**FIGURE 1:**
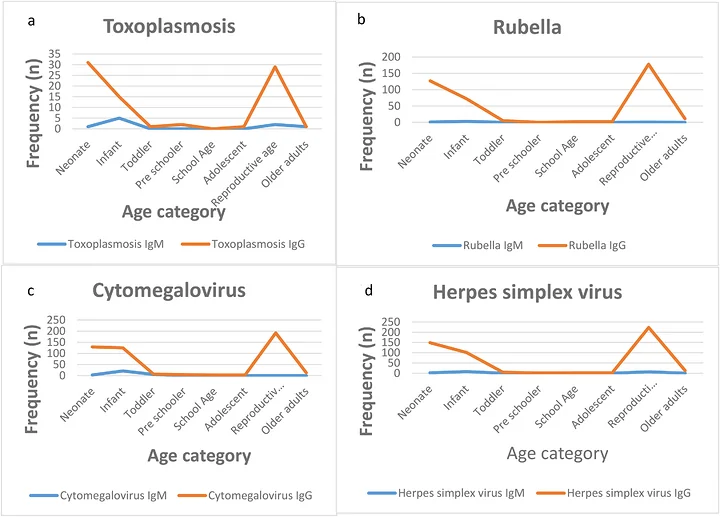
Frequency of IgG and IgM positivity for all *Toxoplasma gondii* (a), rubella (b), CMV (c), and herpes (d) infections across age groups at the JDW National Referral Hospital, Bhutan, 2019–2022. IgG, immunoglobulin G; IgM, immunoglobulin M.

The distribution of TORCH infections varied across different age groups. Both IgG and IgM seropositivity were highest among neonates and infants aged 0–1 year, followed by a decline during the toddler, preschool, school‐age, and adolescent periods, and a subsequent rise in the reproductive age group, as described in Table 1 and Figure 1.

**TABLE 1:** Distribution of *Toxoplasma gondii*, rubella virus, CMV, and herpes immunoglobulin G (IgG) and immunoglobulin M (IgM) seropositivity among children and women tested at the Jigme Dorji Wangchuck (JDW) National Referral Hospital, Bhutan, 2019–2022.

Age group	Toxoplasma gondii		Rubella virus		CMV		HSV		Total
**Pediatric age**	**IgM *(n)***	**IgG *(n)***	**IgM *(n)***	**IgG *(n)***	**IgM *(n)***	**IgG *(n)***	**IgM *(n)***	**IgG *(n)***	
0–28 days (neonates)	1	31	1	127	3	129	2	149	***443***
1–12 months (infant)	5	15	3	72	21	125	8	101	***350***
1–3 years (Toddler)	0	1	0	5	5	7	0	5	***23***
3–6years (preschool)	0	2	0	0	0	4	0	1	***7***
6–12 years (school‐age child)	0	0	0	2	1	3	0	2	***8***
**Teenage females**									
>12–<18 years (adolescent)	0	1	0	2	0	3	0	2	***8***
**Adult women**									
≥18–49 years (reproductive age)	2	29	1	178	0	192	7	223	***632***
>50 years (older adults)	1	1	0	11	0	13	0	13	***39***

Abbreviations: CMV, cytomegalovirus; HSV, herpes simplex virus.

Among the TORCH agents, HSV had the highest IgG seropositivity (81.1%), whereas acute (IgM) infections were most frequently observed with CMV (4.8%). Participants with both IgM and IgG positivity were predominantly seen with CMV infection (4.3%). Overall, the highest positivity was seen in HSV (83.8%) and the lowest in *T. gondii* (14.7%). Table 2 summarizes the seropositivity of TORCH infections among the study participants. The distribution of IgM, IgG, and both IgG/IgM antibody responses is presented separately, along with the overall positivity rate for each infection.

**TABLE 2:** Immunoglobulin G (IgG), immunoglobulin M (IgM), and overall positivity rates of *Toxoplasma gondii*, rubella virus, CMV, and herpes pathogens among children and women tested at the Jigme Dorji Wangchuck (JDW) National Referral Hospital, Bhutan, 2019–2022.

Infection (*n* = 624)	IgM *n* (%)	IgG *n* (%)	Both IgM/IgG *n* (%)	Overall positivity (IgM + IgG) *n* (%)
*T. gondii*	9 (1.4)	83 (13.3)	4 (0.64)	92 (14.7)
Rubella virus	5 (0.8)	405 (64.9)	2 (0.32)	410 (65.7)
CMV	30 (4.8)	486 (77.8)	27 (4.3)	516 (82.7)
HSV	17 (2.7)	506 (81.1)	9 (1.4)	523 (83.8)

Abbreviations: CMV, cytomegalovirus; HSV, herpes simplex virus.

## 4 Discussion

This study provides the first baseline data on the seropositivity of TORCH infections among children <18years and adult female patients tested at the JDW National Referral Hospital, Bhutan. The findings have important implications for maternal and child health programs in the country.

Among the TORCH infections, HSV showed the highest seropositivity rate of 83.8% followed by CMV 82.7%. Among the immunology types, IgG predominates, which is expected due to its lifelong persistence after latent infection and periodic reactivation. A systematic review and meta‐analysis conducted among pregnant women in Turkey demonstrated CMV (94.2%) as the commonest seropositivity detected, followed by rubella (91.2%) and HSV (35.5%) [25]. Compared with the Turkish study, the present findings demonstrate a markedly higher HSV seroprevalence. This difference is likely multifactorial. First, differences in study populations may have contributed, as the Turkish study was restricted to pregnant women, whereas the present study included a broader population comprising both children and non‐pregnant adults, with varying exposure histories. Second, differences in background epidemiology, sexual exposure patterns, and healthcare access between settings may have influenced HSV transmission dynamics. In addition, methodological variations in serological assays and diagnostic thresholds may have also accounted for differences in reported seroprevalence. These factors together may explain the observed discrepancy in HSV seropositivity between the two studies.

Rubella seropositivity was detected in over two‐thirds of cases, predominantly of the IgG type, with only five cases (0.8%) being IgM‐positive. The high IgG rate can be attributed to Bhutan's successful Mumps Measles Rubella vaccination program under the Expanded Program on Immunization (EPI). Rubella‐containing vaccine was first introduced into the routine immunization schedule in 2006 as the measles–rubella (MR) vaccine and was subsequently replaced by the measles–mumps–rubella (MMR) vaccine in 2016 that provides two doses at 9 and 24 months, ensuring long‐term immunity and a decline in recent infections [26, 27]. Through the EPI, all vaccines are given to the eligible population free of cost by the Ministry of Health, Royal Government of Bhutan.

*T. gondii* had the lowest overall seroprevalence (14.7%) among the TORCH pathogens. The relatively low IgG seropositivity suggests lower prior exposure compared with reports from regions such as South America and Africa, where higher seroprevalence has been documented. However, nine individuals (1.4%) were IgM‐positive, indicating possible recent infection. Although few in number, these cases are clinically significant because primary maternal toxoplasmosis during pregnancy can result in congenital infection and adverse fetal outcomes.

The overall seropositivity rates were comparable to those reported in other developing countries, with HSV and CMV emerging as the leading infections [16, 17, 18]. The predominance of IgG seropositivity across all TORCH agents reflects past exposure and possible long‐term immunity, whereas lower IgM positivity suggests limited current infections. Detection of both IgG and IgM in some participants, particularly for CMV (4.3%) and HSV (1.4%), may indicate recent infection in new patients and reactivation or reinfection in older patients.

In the age‐specific analysis, higher IgG seropositivity observed in the 0–1 year age group is likely largely attributable to transplacental transfer of maternal IgG antibodies rather than true infection in the infants themselves, as maternal IgG can persist for up to 12 months. Therefore, IgG positivity in this cohort should be interpreted cautiously and does not necessarily indicate congenital infection. In contrast, IgM positivity in this age group is more suggestive of possible in utero or perinatal infection, although the number of IgM‐positive cases in this subgroup was limited. Seropositivity declined in the toddler, preschool, school‐age, and adolescent groups, likely reflecting waning maternal antibodies and lower early‐life exposure. A subsequent increase in the reproductive age group suggests cumulative exposure over time and highlights ongoing risk of primary infection during pregnancy. These findings underscore the importance of targeted preconception screening and preventive strategies in women of reproductive age to reduce the risk of congenital TORCH infections.

Although maternal infection during pregnancy can lead to adverse outcomes, such as intrauterine infection and congenital anomalies, international guidelines from the Society of Maternal–Fetal Medicine, Royal College of Obstetrics and Gynecology, American College of Obstetrics and Gynecology, do not recommend routine screening for low‐risk pregnancies. Those international societies advise doing TORCH testing only when there are specific clinical indications, such as symptoms of maternal infection, detection of fetal abnormalities in ultrasound, or neonatal signs suggestive of congenital infection [28, 29, 30, 31].

In Bhutan, a similar targeted screening approach is currently practiced. However, operational and health system constraints limit optimal implementation. Currently, TORCH testing is available only at JDW National Referral Hospital, often interrupted by shortages of test reagents. Of the 9240 deliveries reported nationally, 3404 (about 37%) occurred at the JDW National Referral Hospital, indicating that most births take place outside the national referral hospital [24, 32]. Despite Bhutan's high institutional delivery coverage (98%) [32], access to specialized tests like TORCH screening remains centralized, creating inequities for women in remote areas.

At the policy level, the Reproductive, Maternal, Newborn, Child, Adolescent, and Health of the Aged (RMNCAHA) Strategy currently includes routine antenatal screening for syphilis, HIV, and viral hepatitis; however, toxoplasmosis, rubella, and CMV are not yet included. The strategy recommends expanding screening to high‐risk mothers and decentralizing reagent supply to district hospitals. These priorities are aligned with the WHO's call for integrated maternal infection surveillance and the targets of Sustainable Development Goal 3, which aims to reduce maternal and neonatal mortality. Strengthening TORCH surveillance, expanding access to diagnostic testing, enhancing public health education on infection prevention, and establishing a national surveillance system for congenital infections would support early detection, improve equity of care, and advance Bhutan's maternal and child health outcomes.

## 5 Limitations

A key limitation of this study is its retrospective design, based on laboratory records of blood samples from women and children of both sexes. Paired mother–newborn data were not available, preventing differentiation between transplacental maternal IgG transfer and true congenital infection in children. Consequently, the direct impact of maternal TORCH infections on pregnancy and neonatal outcomes could not be assessed. In addition, the study relied on rapid ICTs without confirmatory assays, such as ELISA or IgG avidity testing, which may have resulted in false‐positive IgM results and limited the interpretation of acute infections.

These limitations highlight the need for prospective cohort studies that follow pregnant women and their newborns, incorporate confirmatory serological testing, and evaluate maternal–fetal transmission as well as clinical outcomes associated with TORCH infections in Bhutan.

## 6 Conclusion

This study establishes baseline seroprevalence data for TORCH infections among children (<18 years) and adult females in Bhutan, demonstrating age‐related variations with higher IgG and IgM positivity in neonates, infants, and women of reproductive age. HSV showed the highest overall seroprevalence, whereas CMV demonstrated the highest recent or active infection markers, and temporal fluctuations were observed between 2019 and 2022. These findings provide important evidence to guide maternal and child health policies, including screening and preventive strategies in Bhutan.

## Author Contributions

**Karma Norbu**: conceptualization, methodology, data curation, formal analysis, investigation, writing – original draft. **Sonam Wangzin Rabjay**: conceptualization, methodology, data curation, formal analysis, data analysis, writing – original draft. **Karma Galey**: resources, supervision, investigation, writing the original draft. **Thinley Dorji**: conceptualization, data curation, formal analysis, investigation, methodology, writing – original draft. **Ugyen Chophel**: conceptualization, data curation, formal analysis, data analysis, methodology, writing – original draft. **Jamyang Wangmo**: conceptualization, methodology, data curation. **Binita Rai**: conceptualization, methodology, data curation. **Tshokey Tshokey**: conceptualization, methodology, data curation, writing – original draft. **Yeshey Dorjey**: conceptualization, methodology, data curation, writing – original draft. All the authors read and finalized the final version of the manuscript.

## Funding

The authors have nothing to report.

## Ethics Statement

Ethical approval was obtained from the Institutional Review Board (IRB) of the Khesar Gyalpo University of Medical Sciences of Bhutan (Ref No.: IRB/Approval/PN/2024‐012/1435 dated fourth February 2025). All data were anonymized, and personal identifiers were removed before analysis. Data were stored in a password‐protected computer, and physical copies locked in a cabinet accessible only to the investigators.

## Consent

Informed consent was waived as this was a retrospective review of the laboratory register.

## Conflicts of Interest

The authors declare no conflicts of interest.

## Data Availability

The data that support the findings of this study are available from the corresponding author upon reasonable request.
